# Evidence that a primary anti-viral stimulation of the immune response by OM-85 reduces susceptibility to a secondary respiratory bacterial infection in mice

**DOI:** 10.1186/s13052-018-0569-7

**Published:** 2018-09-26

**Authors:** Giovanni A. Rossi, Wolfgang Bessler, Stefania Ballarini, Christian Pasquali

**Affiliations:** 1 Pediatric Pulmonology and Allergy Units, IRCCS Istituto Giannina Gaslini, Genoa, Italy; 2 OM Pharma SA, A Company of the Vifor Pharma Group, Geneva, Switzerland

**Keywords:** Respiratory infection, Bacterial extract, *Streptococcus pneumoniae*, Influenza virus, Adaptive immunity

## Abstract

**Background:**

Viral respiratory infections may promote bacterial super-infection decreasing the host immune response efficiency. However, using a mice model we recently demonstrated that preventive treatment with the bacterial extract OM-85 reduces the susceptibility to a secondary *Streptococcus* (*S*.) *pneumoniae* infection after influenza virus (I.V.) challenge.

**Methods:**

To better characterize the efficacy of OM-85 against *S. pneumoniae* super-infection, a post-hoc analysis was conducted, comparing efficacy (survival) and morbidity signs (clinical score, body temperature and weight loss) in the OM-85 and the control (BLANC) groups of mice after: a) I.V. infection; b) primary *S. pneumoniae* infection and c) post-I.V. *S. pneumoniae* super-infection.

**Results:**

After a sublethal I.V. dose, all mice stayed alive at day 5 and no differences in morbidity signs were detected between the OM-85 and the BLANC groups. However, OM-85 pretreatment led to a significantly reduction of the viral load in the lung on day 5 post viral infection and, on day 10, reduced neutrophilic inflammation while increasing influenza-specific CD8 + T-cell proportion in the airways. Conversely to viral infection, exposure to *S. pneumoniae* induced a dramatic reduction of survival, with no mice surviving on day 3 post infection in the BLANC group, whereas a partial protective effect was observed in OM-85 pre-treated mice (20% of mice surviving at day 3, and 10% at day 4 and 5). The morbidity data substantiated the survival results. Interestingly, in the “super-infection” study, when mice were exposed to a sublethal I.V. dose followed by a secondary *S. pneumoniae* infection, all mice died by day 4 in the BLANC group. In contrast, in the OM-85 treated group, the survival rate was 70% at day 4 and still 50% at day 5, with positive effects on the clinical scores and on the body temperature already detectable at days 1 and 2.

**Conclusions:**

The efficacy of OM-85 pre-treatment against *S. pneumoniae* super-infection reflects a strong and immediate immune reaction from the host, an event that can be explained in part by a “non-specific” activation of the immune system, a positive “immune effect” of the general OM-85- induced immune response against I.V.

## Background

Viral infections, leading cause of morbidity in the general population, are often relatively mild and frequently confined to the upper respiratory tract [1, 2]. However, they can progress to lower respiratory tract infections (RTI). Under these circumstances, severe disease can develop, requiring hospitalization and potentially leading to death [3].There is a growing body of evidence that viral respiratory infections can promote bacterial super-infection by decreasing the efficiency of bacterial clearance by innate immune cells [4, 5], by damaging the airway epithelial surface and facilitating the bacterial adherence, but also suppressing the adaptive immune response [6]. Severe *Streptococcus* (*S*.) *pneumoniae* infection complicating influenza is the classical example of this sequence of events [3, 7, 8]. The mechanisms increasing the susceptibility to recurrent RTIs overall reflect a poor efficiency of the host defenses [1, 2]. After birth the immune system matures and develops the capacity to effectively control infections, the contact between the host and the environment being an absolute evolutionary necessity [9, 10]. In the recent years, the so called “hygiene hypothesis” has supported the idea that living in cleaner environments increases the risk not only of allergy and asthma, but of also of recurrent RTIs and other respiratory diseases [11–13]. Therefore, treatments based on the stimulation of the immune system by derivatives mimicking the effect of bacteria, as substitute for the “protective” role of infection, has been considered a rational preventive approach [14]. Clinical reports have supported the concept that exposure to bacterial components may influence also the response to a variety of other respiratory pathogens, including viruses [15–19]. In children, treatment with the bacterial extract Broncho Vaxom™ (commonly termed OM-85 in experimental studies) is effective against recurrent RTI reducing not only their frequency and duration, but also their intensity [16–18]. Moreover OM-85 has even an anti-viral effect which mechanisms have been recently characterized in a mouse model of sublethal influenza virus infection [20]. In OM-85 treated animals decreased viral load in lung tissue and increased numbers of influenza-specific CD8+ cytotoxic T-cells in bronchoalveolar lavage fluid were detected. In these mice, OM-85 led additionally to a non-specific maturation of dendritic cells, with overexpression of surface molecules involved in antigen presentation. Concomitantly, a polyclonal B-cell activation with significant increase in the serum IgG levels and trends toward increased IgA and IgG in the airways was also demonstrated [20]. Interestingly, in the “super-infection” study, the susceptibility to a secondary *S. pneumoniae* infection, induced in mice 7 days after the influenza infection, was reduced by OM-85 [20]. Whether the efficacy of OM-85 against *S. pneumoniae* could have been enhanced by the previous influenza virus-induced non-specific activation of the immune response is not known. In a preliminary set of experiments, the efficacy of OM-85 in protecting mice against a primary *S. pneumoniae* was tested, but the results were not reported and not compared with those of the secondary post-influenza *S. pneumoniae* study. The evaluation of these preliminary data, comparing the activity of OM-85 on: a) Influenza infection; b) Primary *S. pneumoniae* and c) Post-influenza *S. pneumoniae* super-infection, was the objective of the present post-hoc analysis.

## Methods

**Fig. 1:**
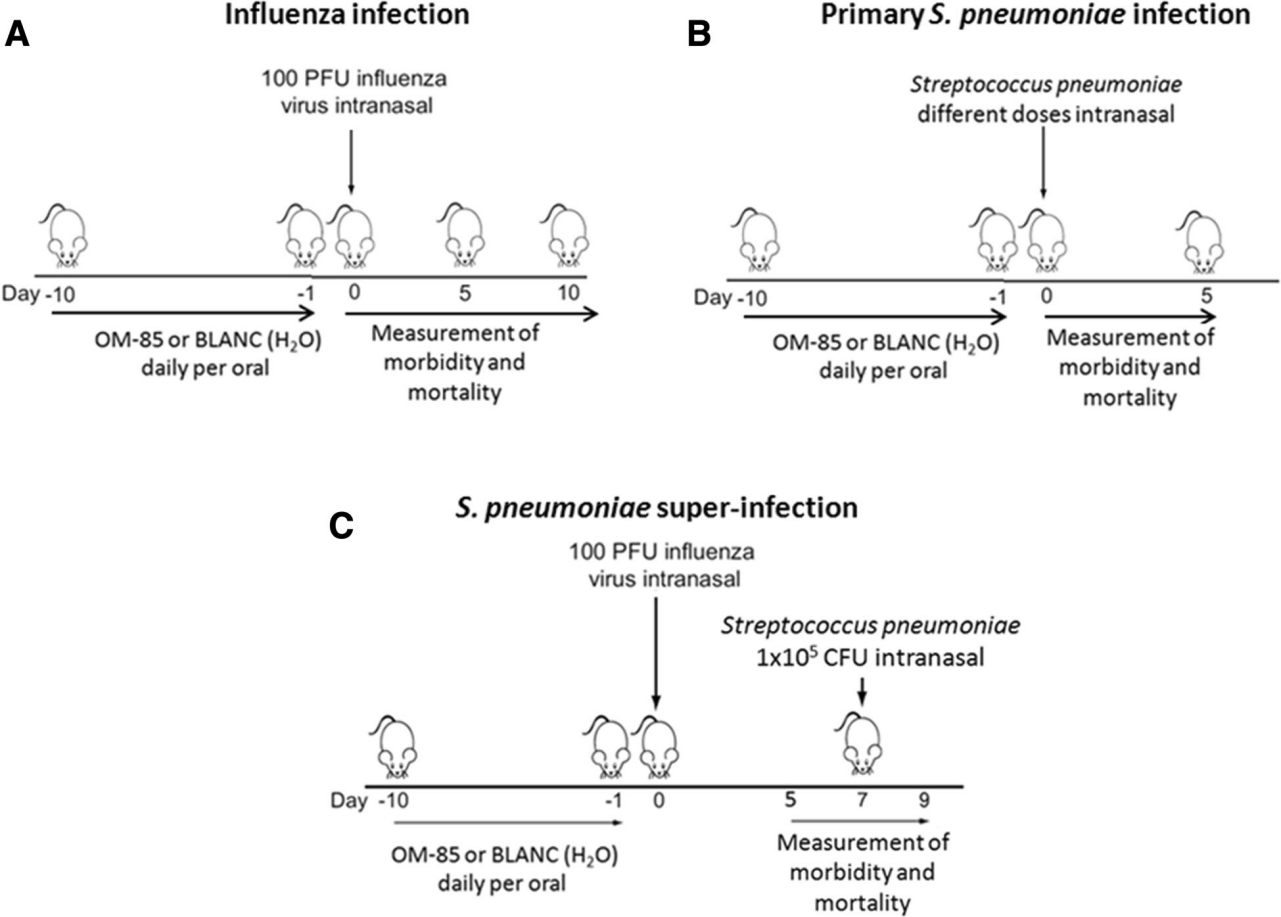
Overview of the experimental conditions in the three studies: **a** “influenza infection; **b** “Primary *S. pneumoniae* infection”; **c**. “*S. pneumoniae* super-infection”

A detailed description of the original study design had been reported in details in the original manuscript [20] and will only be briefly summarized here. In the three studies, “Influenza infection”, “Primary *S. pneumoniae* infection” and “*S. pneumoniae* super-infection”, during the first 10 days, daily gavage of female BALB/c mice aged 8 weeks was performed with 7.2 mg of OM-85-active principle, corresponding to 320 μL of soluble OM-85 concentrate, or its equivalent engineered bioprocess without any bacterial extract content (referred to as “BLANC”) (Fig. 1a, b and c). The number of female BALB/c mice aged 8 weeks included were: A) in “Influenza infection”: 10 mice per group up to day 10 plus 5 mice per group scarified at day 5 for the determination of viral load in the lung,”; B) “Primary *S. pneumoniae* infection”, 5 mice per group (as in the figure) plus another group of 2 × 10 mice of each group for de determination of the bacteremia; C) in “*S. pneumoniae* super-infection”, 10 mice per group up to day 10, plus 5 mice per group sacrified at day 5 for the determination of viral load in the lung. In the “Influenza infection” and “*S. pneumoniae* super-infection” studies, mice received a sublethal dose (100 plaque forming units or PFU) of influenza virus strain PR8 by intranasal administration (Fig. 1a and c). In the “Primary infection” study (Fig. 1b), a preliminary set of experiments was performed to define the number of *S. pneumoniae* lethality. Sixty five mice (13 groups of 5 mice) pre-treated with OM-85 or BLANC for 10 days were infected intranasally with 50 μL of the *S. pneumoniae* bacterial solution samples containing four different CFU doses: Dose A = 1.4 × 10^8^ CFU; Dose B = 7.0 × 10^7^ CFU; Dose C = 3.5 × 10^7^ CFU; Dose D = 1.75 × 10^7^ CFU [20]. In contrast, in the “*S. pneumoniae* super-infection” study (Figure c), *S. pneumoniae* solution was administered intranasally at a lower CFU dose (1.0 × 10^5^) 5 days after intranasal administration of a sublethal dose (100 PFU) of influenza virus strain PR8. Daily measurements of the clinical score (ranging from 1 to 5), weight and body temperature were performed during day 0 to 5 or 10, post influenza virus infection and during day 0 to 5, post *S. pneumoniae* infection and super-infections. Disease score 1 was for a healthy mouse; disease score 2 for a mouse showing signs of malaise, including slight piloerection, slightly changed gait and increased ambulation; disease score 3 for a mouse showing signs of strong piloerection, constricted abdomen, changed gait and periods of inactivity; disease score 4 for a mouse with enhanced characteristics of the previous group, but showing little activity and becoming moribund; disease score 5 for a dead mouse. All the experiments were performed in accordance with the Institutional Guidelines and Swiss Federal and Cantonal Laws on Animal Protection.

### Statistical analysis

Student’s *t* test (unpaired, two-tailed) or a one-way ANOVA was used to calculate significance levels between treatment groups, as indicated.

## Results

### Efficacy of OM-85 on influenza virus infection in mice

**Fig. 2:**
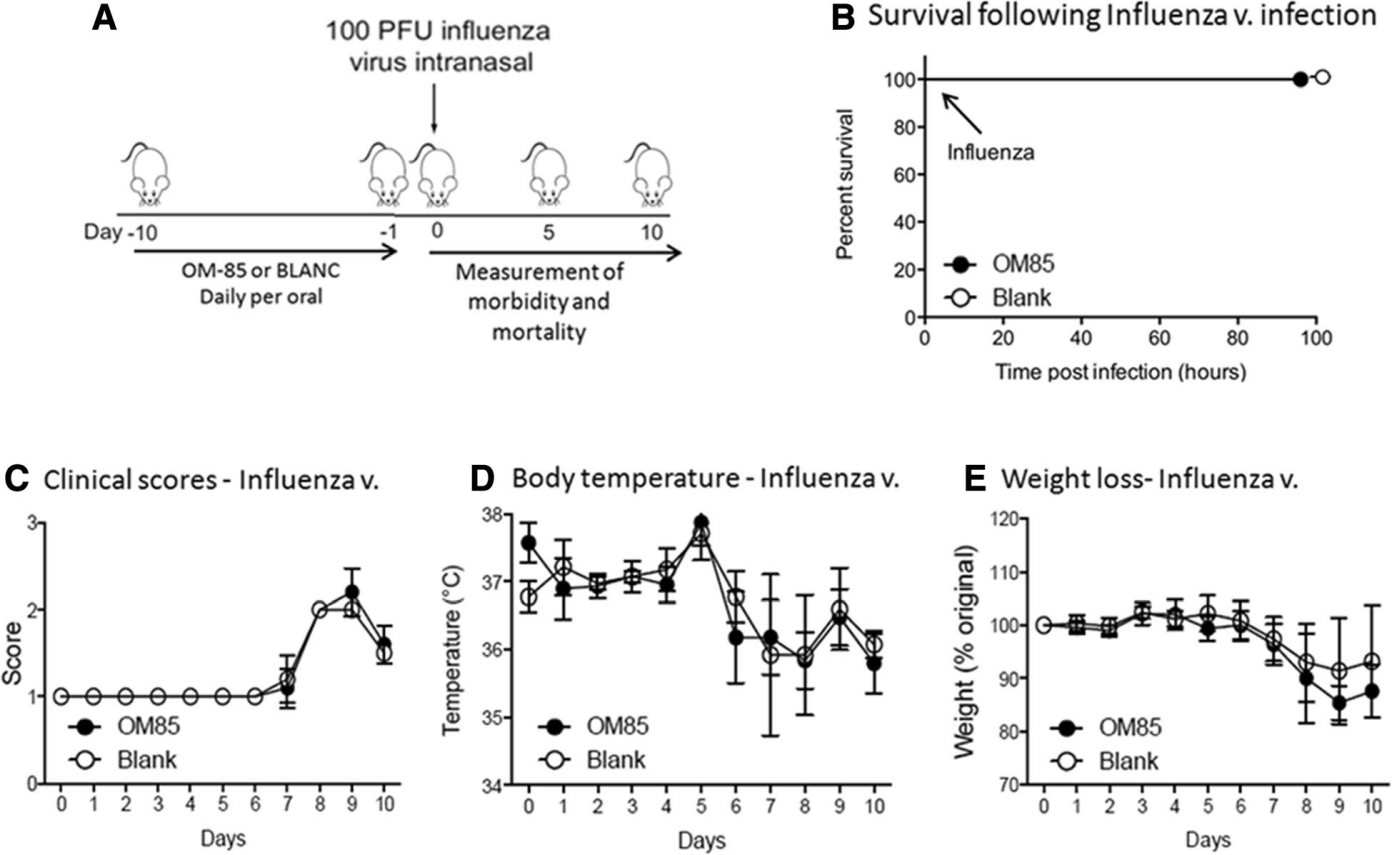
Sublethal influenza virus infection of BALB/c mice. **a** Experimental overview: mice were treated orally with OM-85 or control solution (BLANC) daily for 10 days and then infected by the intranasal administration of a sublethal dose (100 PFU) of influenza virus strain PR8. **b** Survival scheme of mice treated with OM-85 or BLANC and subsequently infected with sublethal dose of influenza virus. The percentage of mice surviving the infection is on the ordinate and the days after the infection is on the abscissa. **c**, **d** and **e** Morbidity of OM-85 or BLANC treated mice and then infected with a sublethal dose of influenza virus. The clinical score, body temperature (expressed in Celsius degrees), and body weight loss (expressed as percentage of the original weight) are on the ordinate and the days after the influenza virus infection on the abscissa. Data are representative of 3 experiments, with 5–10 mice per time point

**Fig. 3:**
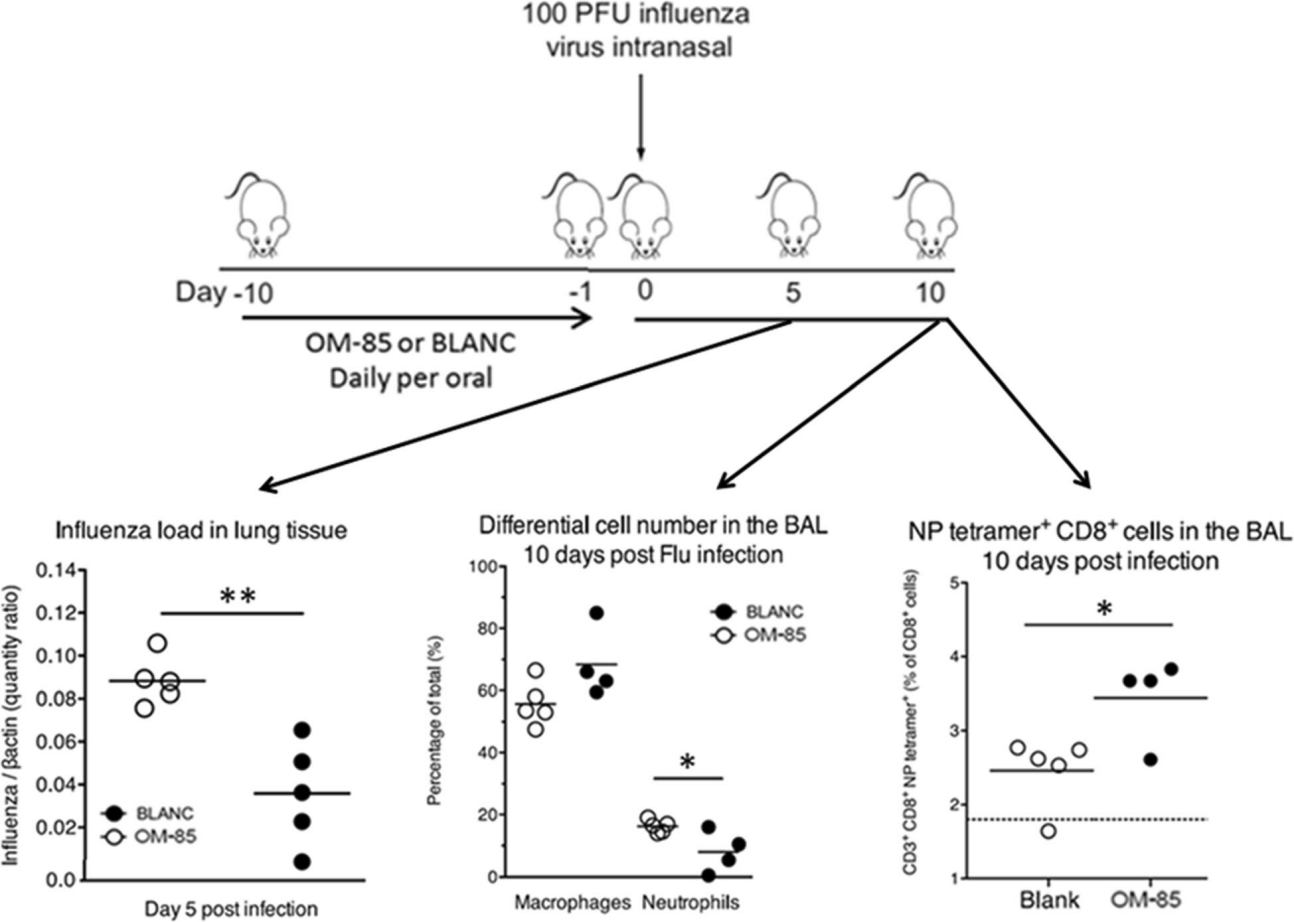
Efficacy of oral preventive treatment with OM-85 on influenza infection. Reduction of the viral load particles in lung tissue on day 5 after infection, and of the bronchoalveolar lavage (BAL) neutrophil and the alveolar macrophages (AM) with increase of the NP-tetramer positive influenza-specific CD8+ T-cell proportion in BAL on day 10 * = *p* < 0.05; ** = *p* < 0.01

In the “viral infection” experiments, and in order to gain insight into whether OM-85 could enhance the anti-viral response, mice were pre-treated orally with OM-85 or BLANC for 10 days. Subsequently, they were infected by an intranasal administration of a sublethal dose (100 PFU) of influenza virus strain PR8 (Fig. 2a). Independently from the type of pre-treatment (OM-85 or BLANC), all mice were alive at day 5 (Fig. 2b) with no morbidity signs as demonstrated by an absence of difference in clinical score, weight loss and body temperature (Fig. 2c, d and e). Conversely, OM-85 pre-treatment significantly lowered the influenza virus lung levels on day 5, the peak of the infection (*p* < 0.01) and reduced the bronchoalveolar lavage (BAL) neutrophilic inflammation on day 10 (*p* < 0.05), an expected reflection of the reduced viral titer seen on day 5(Fig. 3). Interestingly OM-85 increased the NP-tetramer positive influenza-specific CD8+ T-cell proportion in BAL on day 10 (*p* < 0.05), evidencing an enhancement of the adaptive arms of the immune system with consequent improved control of influenza virus infection (Fig. 3).

### Efficacy of OM-85 on *S. pneumoniae* infection in mice

**Fig. 4:**
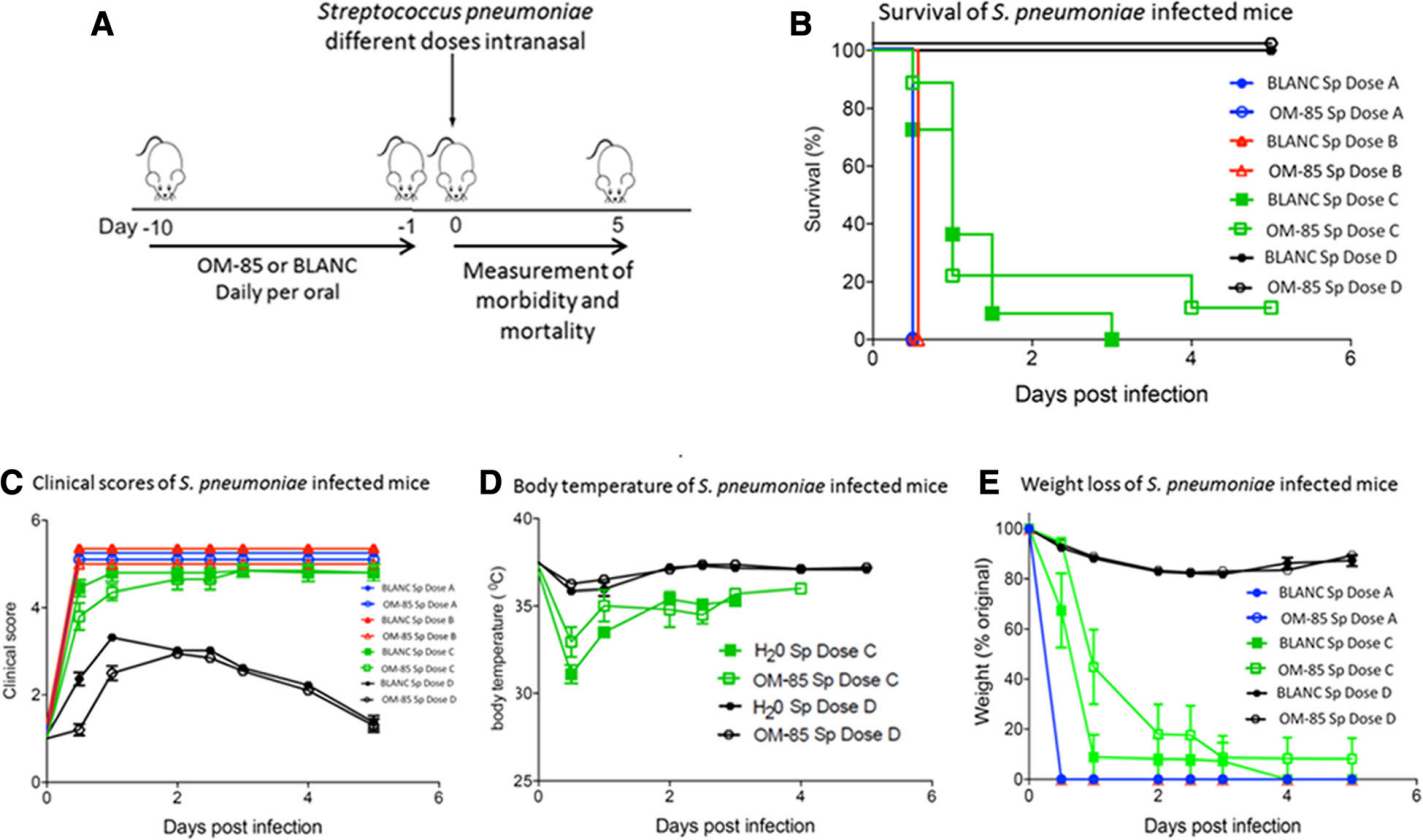
Efficacy of oral treatment with OM-85 on *S. pneumoniae* infection in mice. **a** Experimental overview: BALB/c mice were treated with OM-85 or control solution (BLANC) daily for 10 days and subsequentl infected by intranasal administration of different CFU doses of *S. pneumoniae*. **b** Survival of mice treated with OM-85 or BLANC and then infected with different S*. pneumoniae* CFU doses. The percentage of mice surviving the infection is on the ordinate and the days after the infection is on the abscissa (**c**, **d**, and **e**) Morbidity of OM-85 or control treated mice following *S. pneumoniae* infection. The clinical score, the body temperature and the body weight loss are on the ordinate and the days after the *S. pneumoniae* infection on the abscissa. Data are representative of 3 experiments with 5–6 mice per time point

In the “primary infection” experiments and in order to gain insight into whether OM-85 could enhance the anti-bacterial response, mice were pre-treated orally with OM-85 or BLANC for 10 days and subsequently infected with different doses of *S. pneumoniae* (Fig. 4a). Independently from the type of pre-treatment (OM-85 or BLANC), the two highest *S. pneumoniae* infectious doses (A and B) were highly lethal, with all mice dying within the first 12 h (Fig. 4b). In contrast, all mice treated with dose C (3 × 10^7^) died at day 3 when treated with BLANC, while 20% of those pretreated with OM-85 survived at day 3 and 10% up to day 5. Conversely, all mice survived up to day 5 when exposed to the lowest *S. pneumoniae* dose (D). Despite the low numbers of mice included in the different animal groups and the experiments precludes for a reliable statistical evaluation, the clinical scores reflected the survival data, indicating a protective effect of OM-85 in mice infected by the intermediate *S. pneumoniae* dose (C) (Fig. 4c, d and e).

### Efficacy of OM-85 on “primary” versus “super-infection” studies

**Fig. 5:**
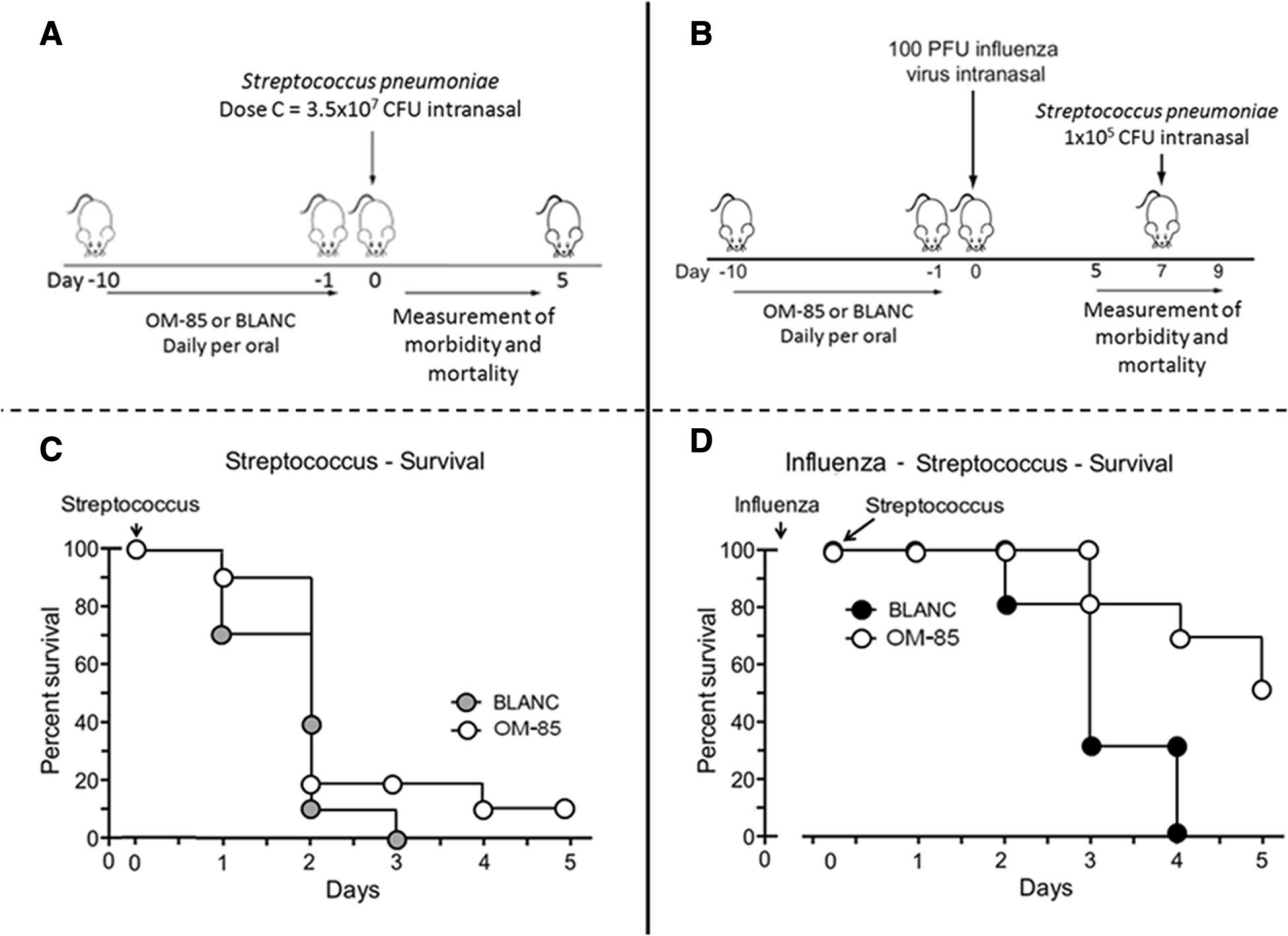
Comparison of the efficacy of oral treatment with OM-85 on *S. pneumoniae* “primary infection” versus “super-infection” (i.e. post-influenza) in mice. **a** and **c**. Survival of mice treated with OM-85 or BLANC subsequently infected with *S. pneumoniae* at 3.5 × 10^7^ CFU dose (Dose C). **b** and **d** Survival of mice treated with OM-85 or control solution (BLANC) and subsequently infected with sublethal dose (100 plaque forming units or PFU) of influenza virus strain PR8, and 7 days later with *S. pneumoniae* at 1.0 × 10^5^ CFU dose. The percentage of mice surviving the infection is on the ordinate and the days after the infection on the abscissa. Data are representative of 3 experiments with 5–6 mice per time point

**Fig. 6:**
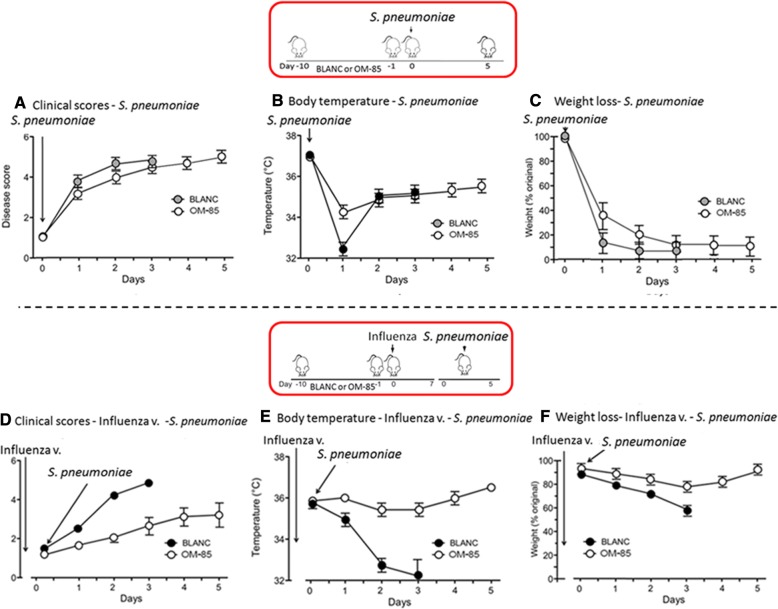
Comparison of the efficacy of oral treatment with OM-85 on the morbidity of *S. pneumoniae* “primary infection” (**a**, **b**, and **c**) versus “super-infection”, i.e. post-influenza infection (**d**, **e** and **f**) in mice, treated with OM-85 or BLANC. The clinical score, body temperature and the body weight loss are on the ordinate and the days after the *S. pneumoniae* infection on the abscissa. Data are representative of various experiments including setting up for the models, with 5–6 mice per time point

In these experiments, mice were exposed to a sublethal influenza infection followed by a secondary, lower *S. pneumoniae* dose (1 × 10^5^) bacterial infection. In contrast to the previous sole “primary infection” study (Fig. 5a and c), the survival rate of mice treated with OM-85improved to a larger extend in the “super-infection” studies (Fig. 5b and d). At day 3, the survival was 80% in the OM-85 group and 30% in the BLAC group; at day 4, all the mice in the BLANC group died whilst the survival was 70% in the OM-85 group and still 50% at day 5. Similar results were detected when clinical score (Fig. 6a and d), body temperature (Fig. 6b and e) and weight loss (Fig. 6c and f) were evaluated. Of note that the positive effects on the clinical scores and on the body temperature were already detectable and evident at day 1 and 2 after the exposure to *S. pneumoniae*, indicative of an immediate immune response of the host to the bacterial infection.

## Discussion

OM-85 is an oral medicine of biological origin: its active principle is an alkaline aqueous soluble extract of 21 bacterial lysates of seven genera isolated originally from human patients suffering from RTIs [21]. Several studies highlighted the capacity of OM-85 to trigger immunomodulatory and protective immune responses against different pathogens and most recently, sequential experimental studies aiming at dissecting its mode of action both in vitro and in vivo, were shared with the scientific community [16, 20, 22–34]. Focusing on infections of bacterial and viral origins, studies in animal or human cell models revealed new insights on the “what” and the “how” such product can modulate the host immune response [24, 26, 33, 34]. Recent literature demonstrate the cell activation, which take place in the gut associated lymphoid tissue, and then migrate to the lung associated lymphoid tissue via the lymphatic circulation [20, 30, 33, 35]. The protective activity of OM-85 against viral infection was further investigated by in situ analysis using human sampling. More precisely, human primary bronchial epithelial cells (BECs) originating from the lung following bronchoscopy using control and disease sampling from donors with RTI [36]. In this last study, the mechanistic anti-viral effect was confirmed by the production of various anti-viral proteins on the surface of BECs, of which, the anti-viral β-defensin. The first relevant finding of the present post-hoc analysis is that susceptibility to lethality occurred within a tight infectious dose window. Despite the fact that an intermediate/low *S. pneumoniae* dose was used, mortality of mice treated with BLANC was higher and concomitant with all morbidity signs. OM-85 treatment appeared to be able to elicit some protective effects; however it was not sufficient to shift the susceptibility to below the threshold of lethality. However, when the mice were exposed firstly to a sublethal influenza infection in the original study [20], pre-treatment with OM-85 led to a more relevant protection against *S. pneumoniae* infection, as shown by improved survival and reduced morbidity. In the original study, OM-85 led to a more relevant protection following a sublethal influenza infection also on *Klebsiella pneumoniae* super-infection [20]. These findings showing an improved resistance to a secondary bacterial infection may be only partially explained by the lower lethality of the *S. pneumoniae* dose used. Yet, an important role is played by the non-specific host immune response against influenza virus infection induced by OM-85 [20]. Indeed and as exemplified earlier in the text, OM-85 can: a) elicit the maturation of dendritic and of B-cells, with upregulation of the surface expression MHC II, CD40, and CD86 [20, 33], b) polyclonally activate B-cells, with a statistically significant increase in IgG levels in serum, and with a trends toward increased IgA and IgG levels in the airways, and c) anti-I.V. antibodies in serum and BAL [20]. The protective effect of the non-specific stimulation of the host defences following viral infection was clearly demonstrated by the observation that, in mice treated with OM-85 (but not in those treated with BLANC), 24 h following the super-infection with *S. pneumoniae,* bacteremia was no longer detected [20]. Considering the important findings that a primary viral infection improves the efficacy of OM-85 to incoming bacterial infections in the host, it is fair to admit some limitations of this post-hoc analysis. One being the fact these data originates mostly from animal origin and that the number of mice used was limited due to the complexity of the experimental methodology and the different in vivo conditions. An additional limitation may also originates from the selection of influenza virus and *S. pneumoniae*, pathogens classically used to study respiratory co-infections in mice [37, 38], but not typically involved in recurrent RTIs in children. Notwithstanding these limitations, it is important to stress that the anti-bacterial efficacy of OM-85 was shown against other bacteria [20, 21]. Concomitantly, the antiviral effects of OM-85 was recently confirmed with mechanistic evidences using human primary lung cells originating from biopsies infected by rhinovirus [36]. The result of our post-hoc analysis support the clinical efficacy of OM-85 BV in the prevention of recurrent RTIs in children and adolescents, shown by meta-analyses and systematic reviews [15, 16, 18, 21]. From the translational aspect, these experimental findings support the concept of using immunomodulatory medicine such as OM-85 BV in the clinical setting of respiratory viral and bacterial super-infections that commonly occur in pediatrics. Although viruses (e.g. rhinovirus, adenovirus, parainfluenza and influenza virus) are the main responsible for RTI, bacterial super-infections sustained by respiratory pathogens (e.g. *S. pneumoniae*, *H. influenzae, S. pyogenes*) are diagnosed in up to 60% of patients with long-lasting (> 10 days) RTI symptoms in this young population [39]. Synergistic effects between viruses and bacteria (commonly influenza virus and *S. pneumoniae*) in the pathogenesis of RTI are also described in the literature [37–40]. Although influenza may be fatal, it is well known that the mortality increases in case of bacterial super-infection [40]. Therefore the described pre-clinical observations explain the clinical efficacy of OM-85 BV in the prevention of RTIs in children and adolescents, the reduction of symptoms severity and duration, as well as of missed days of school, as it is shown in systematic reviews and meta-analysis [15, 16, 18, 21]. The reduction of bacterial complications that are associated with higher morbidity, antibiotic prescription, referral visits to a specialist, and hospitalizations, can also contribute to the control of health care costs [41].

## Conclusions

Considering the recent insights challenging the role of viral versus viral-bacterial co-infections [41–46] in the origin and the pathogenesis of RTI diseases (namely COPD and asthma), the dual and potentially “synergistic” immune response exemplified by OM-85 in these various studies may bring one more additional layer of its mode of action. Since both viruses and bacteria are involved in the pathogenesis of acute and recurrent RTIs, the combined action of OM-85 against both type of pathogens described in the present post-hoc analysis may contribute to explain the positive clinical results obtained in the prevention these common disorders [47].
